# Unmet Healthcare Needs Predict Depression Symptoms among Older Adults

**DOI:** 10.3390/ijerph19158892

**Published:** 2022-07-22

**Authors:** Jonas Eimontas, Goda Gegieckaitė, Olga Zamalijeva, Vilmantė Pakalniškienė

**Affiliations:** Institute of Psychology, Vilnius University, Universiteto Str. 9/1, 01513 Vilnius, Lithuania; goda.gegieckaite@fsf.vu.lt (G.G.); olga.zamalijeva@fsf.vu.lt (O.Z.); vilmante.pakalniskiene@fsf.vu.lt (V.P.)

**Keywords:** older adults, depression, unmet healthcare needs, SHARE

## Abstract

Risk factors for depression in older adults include significant interpersonal losses, increasing social isolation, and deteriorating physical abilities and health that require healthcare. The effects of unmet healthcare needs on depression in older adults are understudied. This study aimed to analyze the association between unmet healthcare needs and symptoms of depression, sleep, and antidepressant medication while controlling for other significant factors among older adults. For this study, we used a multinational database from The Survey of Health, Ageing and Retirement in Europe (SHARE), containing data of individuals aged 50 and older. The final sample used in this research consisted of 39,484 individuals from 50 to 100 years (mean − 71.15, SD ± 9.19), 42.0 percent of whom were male. Three path models exploring relationships between symptoms of depression at an older age and unmet healthcare needs were produced and had a good model fit. We found that unmet healthcare needs were directly related to depression, activity limitations were related to depression directly and through unmet healthcare needs, whereas financial situation mostly indirectly through unmet healthcare needs. We discuss how depression itself could increase unmet healthcare needs.

## 1. Introduction

Healthy aging is paramount to the functioning of our aging society. Depression is the most prevalent mental health issue among older adults that can intervene in healthy aging [1]. A recent meta-analysis found a global prevalence of depression in older adults to be 13.3% [2], while another estimated the prevalence at 28.4% [3]. Depression has a destructive effect on quality of life, physical and psychological health and is a significant obstacle to healthy aging [4,5,6,7]. Risk factors of depression among older adults are often considered to be such prolonged stressors as increasing social isolation, significant interpersonal losses deteriorating health and physical abilities, loss of income, and related financial stress [8,9,10,11]. And while some of these factors cannot be reversed, some areas where intervention is possible receive relatively less attention. One of these areas is the importance of adequate healthcare and the impact unmet healthcare needs can have on older adults’ mental health and depression.

As various health concerns and problems increase with age, there are also growing needs for healthcare services in older age. However, not all healthcare needs are necessarily adequately taken care of by the healthcare system. Unmet healthcare needs can be defined as not receiving an appropriate level of care for the area of significant difficulties [12]. However, in older adult studies unmet healthcare needs are measured in significantly different ways, with some studies measuring a wide range of unmet needs in environmental, physical, psychological, and social areas of functioning [12,13,14,15], while others measured a more specific inability to use needed medical services [16,17,18,19]. Across international surveys and studies of unmet needs of older adults, the cost of the services or the financial situation emerged as one of the most common reasons for unmet healthcare needs [17,19,20]. Long wait times or long distances to travel for services were also common barriers to healthcare services [20]. Health status might also affect unmet health needs in older age as worse health might increase demand for healthcare services that might not be appropriately met and turn into unmet needs.

Unmet healthcare needs in older adults have been found to be associated with deteriorating physical health [21,22]. While the association between unmet healthcare needs and worse physical health seems more straightforward, there is some evidence that unmet needs in older adults might also be associated with worse mental health. In studies measuring unmet needs of older adults in different areas of functioning, it was found that the presence of unmet needs was associated with more depressive symptoms among elderly in nursing homes [23], among low-income older adults [15], and among older outpatients receiving treatment for major depressive disorder [12]. Stein et al. found that German primary care patients aged 75-year-old and older with depression had more unmet needs than those without depression, and unmet needs were also associated with higher depressive symptoms among 75-year-old and older adults in the German general population [14]. However, these studies most often measured unmet needs with The Camberwell Assessment of Need for the Elderly (CANE), which focuses more on social and other services and only partially measures unmet needs in physical healthcare. What is more, several studies that analyzed separate CANE dimensions of unmet needs, found that other areas, like social and psychological unmet needs, but not physical unmet needs were associated with depression severity [12,14,24,25]. While several studies are showing an association between broader unmet needs and depression in older adults, a more detailed analyses revealed contradictory results, as it has been found that unmet needs related to physical health were not associated with depression. The association between depression and unmet needs for medical services specifically remains unclear.

A few studies from Asian countries analyzed the association between depression and unmet healthcare needs, measured as needed but not received medical services within a specific period. Among South Korean adults aged 65 years and older, the group with depression was 1.45 times more likely to have unmet healthcare needs than those without depression [17]. Gao et al. found that middle-aged and older adults in China having depressive symptoms had increased odds of having unmet healthcare needs [19] and the same were found among older Malaysian adults [18]. Only a few studies investigated the association between unmet medical service needs and depression, and there is a lack of these studies in European countries’ settings.

There are several possible mechanisms why unmet healthcare needs and depression might be associated. First, unmet healthcare needs might often lead to deteriorating health, which might affect the quality of life and lead to more depressive symptoms in several ways like adding chronic pain, limiting the ability to move and carry out valued activities, participating in socializing, or even taking care of themselves and their basic needs. Witnessing their deteriorating health might also add to feeling more negative emotions and helplessness about their situation and the future. Second, not receiving needed medical services might mean that needed help is also not received for psychological problems like depression. Untreated depression for prolonged periods has been suggested to be associated with a worse prognosis [26] and has been found to have worse outcomes for depression and related disabilities when treatment is received [27,28]. However, unmet healthcare needs might affect levels of depression not only through deterioration of health but unmet needs by themselves might be significantly stressful, adding to psychological distress. As a few authors have suggested [15,29], recognizing that a person has a medical difficulty that needs to be addressed but not being able to get medical services for it might lead to feelings of frustration and anger as well as feelings of personal helplessness, decreased control and competence which in turn might increase levels of depression. Finally, depression might also have an effect on unmet healthcare needs and reinforce a vicious cycle. For example, depression’s negative effect on physical health might increase healthcare needs and in turn unmet needs and it has been also suggested that depression-caused sense of helplessness, reduced motivation, and lower cognitive abilities might interfere with the ability to effectively seek needed healthcare services [17,18].

Depression significantly alters healthy aging, and earlier findings suggested that unmet healthcare needs can play a role in the development and persistence of depression. However, research findings have been inconclusive. It has been found that unmet healthcare needs are associated with depression in older adults, but most of the studies have focused on the needs in general areas of functioning and more detailed analyses found that unmet needs related to physical health were not associated with depression. The association between not receiving needed medical services and levels of depression remains unclear, especially in the general population of older Europeans. Different healthcare systems also might affect unmet needs and how they are related to depression, most of the previous studies were focusing on one country’s population, limiting the ability to generalize findings to different healthcare settings. This study aimed to analyze the association between unmet healthcare needs and depressive symptoms, as well as sleep and antidepressant medication, while controlling for significant factors in a large-scale sample of older adults in 28 European countries and Israel.

## 2. Materials and Methods

### 2.1. Data and Study Design

For the purpose of this study a multinational database from The Survey of Health, Ageing and Retirement in Europe (SHARE) containing longitudinal micro-level data from interviews conducted with individuals and their partners aged 50 and older was used [30]. SHARE data is collected every two years in 28 European countries as well as Israel. It includes information on socioeconomic situation and health related measures. The data was drawn from SHARE wave 7 (data collected in 2017; [31]) and wave 8 (data collected in 2019–2020; [32]). SHARE wave 7 focused on the life course perspective and included information about retrospective healthcare [33], while wave 8 primarily focused on the current functioning of respondents and included information on current mental health status [34]. The final sample used in this study consisted of 39 484 subjects that participated in both wave 7 and 8. Participants’ age ranged from 50 to 100 years (mean − 71.15, SD ± 9.19), and 42 percent of them were male.

### 2.2. Variables

#### 2.2.1. Current Mental Health Outcomes

Information on respondents’ current mental health was derived from the SHARE wave 8 dataset.

Depressive symptoms. EURO-D scale was developed based on Geriatric Mental State Examination [35] and is a frequently used self-report instrument to estimate the presence of depressive symptoms among older populations. The scale was validated and is widely used in cross-country research [36]. EURO-D covers 12 symptoms: sadness, pessimism, suicidality, self-blame, trouble sleeping, loss of interest, irritability, appetite changes, fatigue, difficulty concentrating, loss of enjoyment, and tearfulness. The items are scored 0 (symptom not present) or 1 (symptom present), and summed scores range from 0 to 12, with higher scores indicating a greater degree of depression.

Use of medication. In addition to the assessment of depression symptoms, two mental health outcomes were included in the analysis–the use of medication for mental health and related issues. The use of anxiolytic and antidepressant medication naturally implies diminished mental health or even an acute mental illness. Moreover, sleep disturbances have been identified as comorbid with mental disorders. Sleep complaints are present in the majority of depression cases and are a good predictor of major depression [37]. Moreover, sleep disorders are more prevalent in later life. They are linked with decreased well-being and may be a sign of an undiagnosed mental health condition [38,39], thus, the use of sleep medication is a reliable indicator of mental health status among older adults. During the interview, respondents were asked to indicate what medications they currently take at least once a week from a list of 15 items, including medication for “sleep problems” and “anxiety or depression”. The answers were coded as “yes” (1) or “no” (0).

#### 2.2.2. Unmet Healthcare Needs

Information on respondents’ unmet healthcare needs was derived from the SHARE wave 7 and wave 8 datasets.

Unmet healthcare needs were measured using six indicators. Four of those represent unmet healthcare needs over the course of respondent’s life, including childhood, youth, and adult life. The data representing lifetime experience of difficulties fulfilling healthcare needs was extracted from SHARE wave 7 data set. During that interview, respondents were asked to report whether or not they ever (1) needed to see a doctor but did not because they could not afford it, (2) needed to see a doctor but could not because they had to wait too long for an appointment, (3) postponed a dentist visit to help them keep their living costs down, (4) foregone taking medication which they could not afford because of cost. The other two items provide information on the recent barriers to healthcare, i.e., measure unmet healthcare needs over the last 12 months, and were extracted from SHARE wave 8 dataset. To collect this information, respondents were provided with a list of services, which included care from general practitioners, specialist physicians, dental care, optical care, homecare, paid home help as well as medication. Then they were asked to indicate whether they had to forgo any of these healthcare services from the list (1) because of the cost they would have to pay or (2) due to the reason these services were unavailable or not easily accessible. They could also include other related services not mentioned on the list. The answers were coded as “yes” (1) if the respondent indicated at least one service they had to forgo.

#### 2.2.3. Other Predictive Variables

Older adults are more likely to have long-term health conditions [40] that are linked to the greater number of limitations in everyday functioning and a subsequently increased risk of mental health problems [41,42], thus, physical health should be taken into account when analyzing the mental health of older adults. Global Activity Limitation Index (GALI) is a one-item measure used in SHARE to assess overall functioning and long-term restrictions experienced by respondents [43]. Respondents are asked to evaluate to what extent they have been limited in everyday activities due to health problems over the last six months by choosing one of 3 answer options: severely limited, limited but not severely and not limited. For the purpose of this analysis, results were recoded into two categories: “limited” and “not limited”.

A substantial part of indicators regarding unmet healthcare needs included in this research have a financial aspect, i.e., failure to get the healthcare services was due to expenses subjects found challenging to pay. Moreover, there are reports suggesting an association between financial disadvantage and the risk of mental health issues among older adults [11], thus, it was reasonable to include a measure representing the financial situation. Since respondents in this sample were from various countries, the factual monetary income per household member may not be a comparable indicator. Therefore, we considered a subjective measure of the financial situation to be more appropriate for this analysis. Even though both partners are interviewed in SHARE, the economic aspects are measured on a household level, i.e., only one household representative has respective data regarding the household’s financial situation. To measure their financial situation, respondents were asked to consider their household’s total monthly income and evaluate whether their household is able to make ends meet with great difficulty, with some difficulty, fairly easily or easily.

Both GALI and subjective financial situation estimates were extracted from SHARE wave 7 dataset.

### 2.3. Statistical Analysis

In this study IBM SPSS 27 (IBM, Chicago, IL, USA) and Mplus 8.2 [44] were used for data analysis. At the beginning confirmatory factor analysis was conducted to test whether it was possible to have one latent construct named unmet healthcare needs that included all six selected measurement variables described above. After finalizing a unidimensional model of unmet healthcare needs, other three path models were tested. All three path models included the latent variable indicating unmet healthcare needs. These models also included use of medication for sleep problems, use of medication for anxiety or depression, and depressive symptoms as outcome variables; age and gender as controlled variables. Two path models included Global Activity Limitation Index and only one included a subjective measure of financial situation. Considering that the majority of variables in the path models were binary or categorical, WLSMV (Weighted Least Square Mean and Variance Adjusted) estimator and theta parameterization were applied. Model fit was evaluated based on root mean square error of approximation (RMSEA) index, standardized root mean square residual (SRMR), comparative fit index (CFI), and Tucker-Lewis index (TLI) results. Since χ^2^ criterion is sensitive to the sample size [45], it was not used in the goodness of model fit assessment. However, the difference between models was focused on the change in χ^2^ test. Based on the common practice, the model is considered well-fitting when RMSEA is smaller than 0.06, SRMR is smaller or close to 0.08, CFI and TLI are close to 0.95 or greater [46]. These fit criteria are also suitable for models with dichotomous variables [47]. Results are considered significant at *p* ≤ 0.01.

## 3. Results

### 3.1. Descriptive Analysis

The average depression symptoms, measured by EURO-D, score in the sample was 2.45 (SD ± 2.26) out of a possible 12. A total of 7.9% (N = 3097) of subjects included in the analysis reported using medication at least once a week for sleep problems, and 6.4% (N = 2540)—medication for anxiety or depression. Descriptive characteristics of unmet healthcare needs indicators and a subjective assessment of the financial situation and experienced limitations performing daily tasks are presented in Table 1.

### 3.2. Path Analysis

In this study we hypothesized that the unmet healthcare needs could be a unidimensional construct combined from six observed variables: (1) could not afford to see a doctor when needed, (2) could not see a doctor due to long wait for appointment, (3) postponed dentist visit due to the cost, (4) forgone taking medication due to the cost, (5) forgone healthcare service due to cost over the last 12 months, and (6) forgone healthcare service due to unavailability over the last 12 months. Prior to performing a path analysis, a confirmatory factor analysis (CFA) of unmet healthcare needs was conducted. The unidimensional model was estimated for the whole sample. This initial CFA model did not have satisfactory fit (χ^2^ 9 = 1747.91, *p* < 0.001; CFI = 0.91; TLI = 0.84; RMSEA = 0.07; SRMR = 0.11). Thus, the model was adjusted according to the suggested modification indexes. One correlation between residual variances was included in the model: between the residual variance of items forgone healthcare service due to cost over the last 12 months, and forgone healthcare service due to unavailability over the last 12 months. The final CFA model with this correlation had a good model fit (see Table 2). Factor loadings for the latent construct ranged from 0.41 to 0.87. Thus, the model of CFA suggested that selected unmet healthcare needs indicators could represent a unidimensional construct for the whole sample.

**Figure 1:**
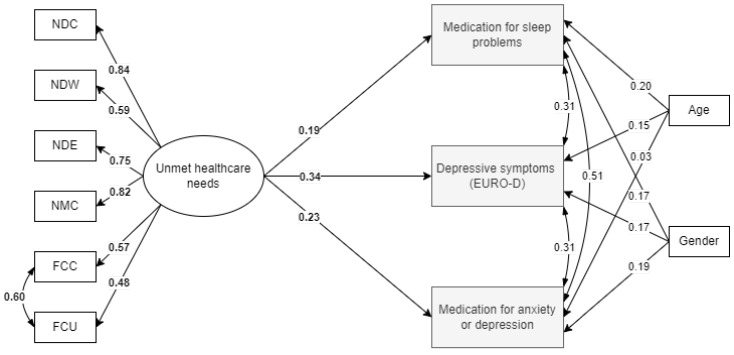
Model A results. Note. All estimates presented are significant at level *p* < 0.001. NDC, didn’t see a doctor due to cost (lifetime); NDW, didn’t see a doctor due to long wait for appointment (lifetime); NDE, postponed dentist visit due to cost (lifetime); NMC, forgone taking medication due to cost (lifetime); FCC, forgone healthcare services due to cost (last 12 months); FCU, forgone healthcare services due to unavailability (last 12 months).

After testing the latent construct three path models were evaluated. In all three models age and gender were included as controlled variables since older age and female gender are considered significant risk factors for depression in later life [48]. The first path model aimed to test whether the latent unmet healthcare needs construct will predict all three current mental health outcomes, including the level of depressive symptoms, use of sleep medication, and use of antidepressants and/or anxiolytics. This model, referred to as Model A in Table 2. Model A fit the data well and results of this analysis suggested that unmet healthcare needs significantly predict all three mental health outcomes (see Figure 1). This suggests that unmet healthcare needs, regardless of when they were unmet, may significantly increase the chance of the use of medication for sleep problems, medication for anxiety and/or depression as well as increase the chance of having higher rates of reported depressive symptoms later in life.

**Figure 2:**
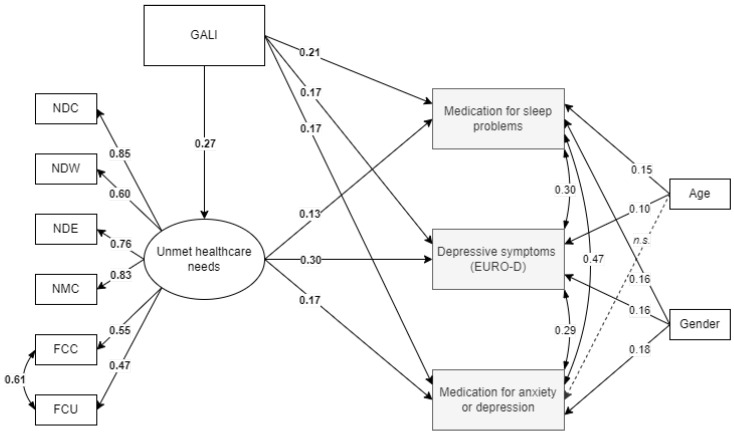
Final Model B results. Note. All estimates presented are significant at level *p* < 0.001. GALI, Global activity limitation index; NDC, didn’t see a doctor due to cost (lifetime); NDW, didn’t see a doctor due to long wait for appointment (lifetime); NDE, postponed dentist visit due to cost (lifetime); NMC, forgone taking medication due to cost (lifetime); FCC, forgone healthcare services due to cost (last 12 months); FCU, forgone healthcare services due to unavailability (last 12 months); n. s., not significant.

The second path model (referred to as Model B in Table 2) tested whether the unmet healthcare needs could still predict mental health outcomes when assessment of overall limitations, represented by Global Activity Limitation Index (GALI), was entered into the model. The role of chronic health conditions and subsequent limitations have been shown to play a considerable role in late-life depression and well-being. Thus, the inclusion of GALI could lessen the predictive value of unmet healthcare needs. Subjects with missing data on GALI were excluded from the analysis. The results of initial Model B show that this model did not fit the data well. Thus, modification indexes suggested to add a regression path from GALI to the latent variable of unmet healthcare needs. Results of this final Model B with this regression path included (see Figure 2) fit the data well (see Table 2). The model suggests that the Global Activity Limitation Index significantly predicts all three mental health outcomes as well as unmet healthcare needs (Est = 0.27). These results show that individuals who experienced long-term limitations in their everyday life due to health issues had a greater probability of using medication for sleep and for mental health-related issues and were more likely to report more depression symptoms. The final model also supports the idea that the presence of limitations increases the possibility of healthcare needs not being fully met.

**Figure 3:**
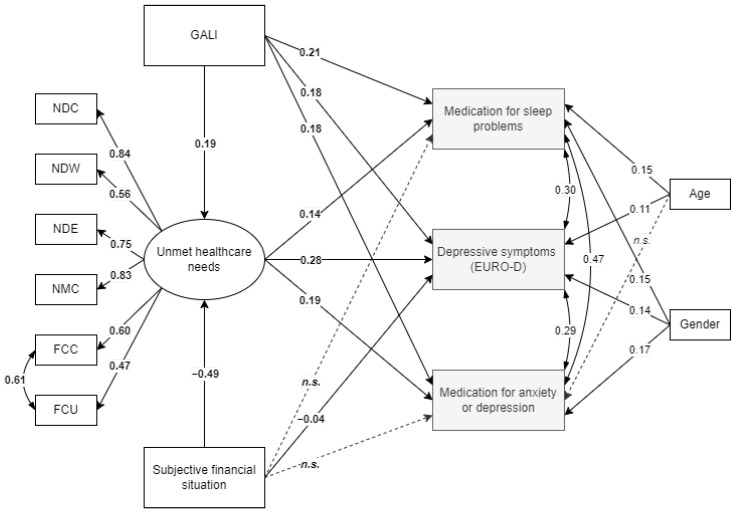
Final Model C results. Note. All estimates presented are significant at level *p* < 0.001. GALI, Global activity limitation index; NDC, didn’t see a doctor due to cost (lifetime); NDW, didn’t see a doctor due to long wait for appointment (lifetime); NDE, postponed dentist visit due to cost (lifetime); NMC, forgone taking medication due to cost (lifetime); FCC, forgone healthcare services due to cost (last 12 months); FCU, forgone healthcare services due to unavailability (last 12 months); n.s., not significant.

Considering that socioeconomic disadvantage has been shown to have a negative impact on various aspects of an individual’s functioning, including mental health, we decided to test one more model that included a subjective measure of financial situation. As mentioned previously, only one household representative, out of two interviewed, provided information regarding the household’s financial situation. It was not considered reasonable to impute missing data in this situation, thus, respondents that had missing data on the subjective financial situation variable were excluded from the analysis. Initial model, where subjective financial situation was added as a predictor of all mental health outcomes (referred to as Model C in Table 2), failed to fit the data. A review of modification indexes showed that a path between subjective financial situation and unmet healthcare needs could be added. This path is consistent with previous literature analysis showing that individuals with less financial resources face more difficulties in having their healthcare needs met, thus, this modification was implemented. The final Model C had a good fit to the data (see Table 2) and showed that out of three mental health outcomes, subjective financial situation predicted only the level of depressive symptoms (see Figure 3). The estimate value of the path between subjective financial situation and depression symptoms was significant but at the same time very small (Est = −0.04) and could even be considered negligible. While the path between subjective financial situation and the latent unmet healthcare needs factor had a moderate significant estimate and showed that the more difficult it was for the individual to make ends meet, the greater the probability of unmet healthcare needs. All other paths introduced in previous models remained significant in the final Model C.

In all three models, the use of medication for sleep problems and anxiety/depression were correlated. Also, the use of medication was correlated with depressive symptoms. In all tested models, age and gender of the participants were controlled by including them as predictors of all three mental health outcomes. Summing up the role of controlled variables, results show that older age predicts higher scores in depressive symptoms and a greater probability of sleep medication use. At the same time, gender predicts all three mental health outcomes and indicates that women are more likely to report more depression symptoms and use medication for sleep disturbances, anxiety, or depression.

## 4. Discussion

The population’s median age in developed societies has been steadily rising for more than five decades. The prevalence of health issues increases with age, and this is directly related to the amount of healthcare services needed. Our study has used data of SHARE project waves 7 and 8 to analyze the relationship between unreceived health treatment and symptoms of depression in older residents of Europe and Israel.

In our sample, 2.7–7.2% of participants reported not receiving needed medical services in their past, and 6.5–8.1% reported having unmet healthcare needs in the last 12 months. These levels of unmet healthcare needs are comparable to the ones found in the previous surveys done on the general population in European countries [20]. Postponing dentist’s visits in the past and forgoing healthcare services in the last 12 months due to cost were the most common reasons in our sample for unmet healthcare needs, similar to what was found in previous studies [17,19,20]. However, unavailability and long wait times were almost as common, and more participants indicated they had not visited a doctor in the past because of the long wait time than because of the cost. Reasons for unmet healthcare needs might vary across European countries because of different healthcare systems. In countries with a public healthcare system, wait times or unavailability might become more relevant than cost. However, private medical services with shorter waiting times might still be available for out-of-pocket fees [49]. Therefore, cost might still be a relevant reason for unmet medical needs.

Our study has found that present depressive symptoms were related to unmet healthcare needs earlier in life and recently while controlling for age and gender, financial situation, and functional limitations in daily activities experienced due to health issues. We included the usage of medication for depression, anxiety, and sleep problems as additional mental health outcomes related to depression and found a significant association between medication use and depression symptoms. Previous studies on older adults, which focused on the broader definition of unmet needs, found a relationship with depression [12,14,15,50]. However, some studies found a stronger relationship existed with unmet psychological or social needs rather than physical ones [12,14]. Unmet care needs measured in our study were exclusively focused on medical services, not excluding possible mental health services, and demonstrate more explicitly than in previous studies that there is an association between depression and unmet needs in received healthcare services. As discussed before, there is a number of ways in which unmet healthcare needs might lead to stronger depressive symptoms: worsening quality of life due to deteriorating health, added stress of not receiving needed services as well as increased negative emotions like frustration, helplessness, and anger. Previous unmet healthcare needs for depression might also exacerbate or make symptoms more resistant to treatment later [27,28]. Unmet healthcare needs may also contribute to greater treatment burden–self-management of the disease, interaction with doctors and complex healthcare system, and other issues become difficult for the patient to manage. Treatment burden has been shown to cause emotional distress [51] and has a negative effect on the well-being [41] and social functioning [52] of older adults suffering from long-term health conditions. Unmet healthcare needs and depression might also have a self-reinforcing association. Existing depression symptoms might increase unmet needs because of worsening health as well as an increased sense of helplessness, fatigue, and problems concentrating and making decisions interfering with successfully seeking medical help.

Based on previous research highlighting the role of long-term health issues and financial disadvantage in the mental health of older adults, measures of the overall assessment of limitations in everyday functioning and subjective financial situation were gradually introduced into the analysis. We found that limitations due to health issues had a direct effect on depression and usage of medications and a similarly strong association with unmet healthcare needs, suggesting that health issues can also indirectly explain the level of depressive symptoms through increased unmet healthcare needs. Subjective financial situation had only a weak association with depression symptoms and a much stronger association with unmet healthcare needs. As unmet healthcare needs in this study were measured as services not received because of lack of financial resources and/or having to wait for an extensive period, the association between worse financial situation and increased unmet needs is expected. People with a worse financial situation might not be able to pay for needed medical services, and it could influence the importance of waiting times as in some European countries it might be possible to access private services quicker by paying out of pocket instead of waiting for the public healthcare services. Many studies found that socioeconomic status was related to depression [11,53,54,55]. However, the relationship between the two is controversial, and it has been suggested that the association could be explained by other underlying factors contributing to lower socioeconomic status [53,54,56]. Low socioeconomic status may affect depression in older adults through various other factors. Our study results demonstrated that financial strain could lead to increased unmet healthcare needs, leading to more depressive symptoms.

The results of this study demonstrate the association unmet healthcare needs have with mental health in older adults. It is known that unmet healthcare needs are associated with worse physical health outcomes down the line [21,22]. However, not much attention has been drawn to the possible effects unmet healthcare needs might also have on mental health as well as depression, possibly interfering with getting adequate medical care. The impact of delayed needed healthcare for older adults is especially relevant after a few years of COVID-19 pandemic when part of the population has postponed seeking medical help due to infection concerns or faced additional obstacles because medical services were limited due to social distancing or other restrictions [57,58]. This might have been especially relevant for older adults as they were the most vulnerable group to the risks of COVID-19 while also being a population with higher healthcare needs. Healthcare providers working with older adults should be aware of the effects unmet healthcare needs might have not only on physical health but also on their mental state. When working with older adults with depressive symptoms, it might be important to address possible unmet healthcare needs and, on the other hand, consider and address how depression might affect their ability to get needed healthcare services. In fact, studies on relevant internet-based interventions for depressed older adults have been in development [59] and could potentially help fill the gap of unmet mental healthcare needs.

### Limitations

Despite the major advantage of having a large multicultural sample of older adults who were enrolled in the study using probability sampling, this study has its limitations that need to be addressed. One of the issues was that the assessment of the financial situation was limited to only one respondent within a couple, and it is more likely that information on financial aspects was provided by a partner most engaged in the household finances, thus, the data was not missing at random. Secondly, there were limited details on specifics of the unmet healthcare needs. Despite covering topics of financial struggles and issues of unavailability or accessibility, there was no information on whether or not these forgone services included psychological or psychiatric help. Further research on the exploration of the unmet psychological needs is required to gain additional insight into the role of unmet healthcare needs in mental health among older adults. While a large multicultural sample is a strength of our study, future research could also test the associations found in this study in different regions and countries to understand the possible effects of different healthcare systems and cultural aspects. While some limitations need to be addressed when interpreting results, this study provides insight into the importance of timely healthcare services for healthy aging.

## 5. Conclusions

In conclusion, unmet healthcare needs during life are strongly associated with symptoms of depression in older adults. More long-term limitations in everyday life due to health issues also predicted depressive symptoms and the use of medication for sleep and mental health-related issues directly and through unmet healthcare needs. The poor financial situation is moderately related to unmet healthcare needs. However, the path between financial situation and depressive symptoms could be considered negligible. Finally, this study demonstrated that understanding how unmet healthcare needs are associated with depression in older age might benefit from studies investigating indirect paths with other common problems older adults face. Moreover, these results can be beneficial for health practitioners and policymakers because they show that neglected healthcare needs may diminish not only the physical but also the mental health of older adults. This relationship is yet another encouragement for the policy makers to look for ways of developing healthcare systems where timely access to quality healthcare is a priority. Mental health practitioners working with depressed older adults should consider that unmet healthcare may contribute to increased depression symptoms. Facilitating and guiding the client towards fulfilling healthcare needs could be needed because depressive symptoms may also hinder them from effectively seeking and receiving it.

## Figures and Tables

**Table 1:** Descriptive characteristics of predictive variables.

Variable	% (N)	Variable	% (N)
Didn’t see a doctor due to cost (lifetime)	4.4 (1369)	House hold is able to make ends meet…	
Didn’t see a doctor due to long wait for appointment (lifetime)	6.0 (1860)	… with great difficulty	12.1 (3283)
Postponed dentist visit due to cost (lifetime)	7.2 (2264)	… with some difficulty	27.6 (7489)
Forgone taking medication due to cost (lifetime)	2.7 (842)	… fairly easily	29.5 (8003)
Forgone healthcare services due to cost (last 12 months)	8.1 (3189)	… easily	30.7 (8335)
Forgone healthcare services due to unavailability (last 12 months)	6.5 (2572)	Reported limitations due to health problems (GALI)	45.8 (18,066)

**Table 2:** Model fit information.

	N	χ^2^ (df)	RMSEA [90% C.I.]	CFI	TLI	SRMR
CFA (initial)	44594	1747.914 *** (9)	0.066 [0.063–0.068]	0.906	0.843	0.113
CFA (final)	44594	72.800 *** (8)	0.013 [0.011–0.016]	0.996	0.993	0.022
Model A	44594	1152.452 *** (35)	0.027 [0.025–0.028]	0.965	0.946	0.049
Model B (initial)	39484	2850.845 *** (41)	0.042 [0.040–0.043]	0.908	0.858	0.073
Model B (final)	39484	1329.730 *** (40)	0.029 [0.027–0.030]	0.958	0.933	0.061
Model C (initial)	27110	5095.106 *** (46)	0.064 [0.062–0.065]	0.760	0.624	0.175
Model C (final)	27110	1040.496 *** (45)	0.029 [0.027–0.030]	0.953	0.924	0.057

Note. *** *p* < 0.001.

## Data Availability

This paper uses data from SHARE Waves 7 and 8 (DOIs: 10.6103/SHARE.w7.800, 10.6103/SHARE.w8.800), see Börsch-Supan et al. (2013) for methodological details.
